# Brain pathology recapitulates physiology: A network meta-analysis

**DOI:** 10.1038/s42003-021-01832-9

**Published:** 2021-03-08

**Authors:** Thomas J. Vanasse, Peter T. Fox, P. Mickle Fox, Franco Cauda, Tommaso Costa, Stephen M. Smith, Simon B. Eickhoff, Jack L. Lancaster

**Affiliations:** 1 Department of Psychiatry, University of Wisconsin-Madison, Madison, WI USA; 2 Research Imaging Institute, University of Texas Health Science Center at San Antonio, San Antonio, TX USA; 3 Department of Radiology, University of Texas Health Science Center at San Antonio, San Antonio, TX USA; 4 South Texas Veterans Health Care System, San Antonio, TX USA; 5 FocusLab and GCS-fMRI, University of Turin and Koelliker Hospital, Turin, Italy; 6 Wellcome Centre for Integrative Neuroimaging (WIN FMRIB), Oxford University, Oxford, UK; 7 Institute of Systems Neuroscience, Medical Faculty, Heinrich Heine University Düsseldorf, Düsseldorf, Germany; 8 Institute of Neuroscience and Medicine, Brain & Behaviour (INM-7), Research Centre Jülich, Jülich, Germany

**Keywords:** Diseases of the nervous system, Cognitive neuroscience

## Abstract

Network architecture is a brain-organizational motif present across spatial scales from cell assemblies to distributed systems. Structural pathology in some neurodegenerative disorders selectively afflicts a subset of functional networks, motivating the network degeneration hypothesis (NDH). Recent evidence suggests that structural pathology recapitulating physiology may be a general property of neuropsychiatric disorders. To test this possibility, we compared functional and structural network meta-analyses drawing upon the BrainMap database. The functional meta-analysis included results from >7,000 experiments of subjects performing >100 task paradigms; the structural meta-analysis included >2,000 experiments of patients with >40 brain disorders. Structure-function network concordance was high: 68% of networks matched (pFWE < 0.01), confirming the broader scope of NDH. This correspondence persisted across higher model orders. A positive linear association between disease and behavioral entropy (p = 0.0006;R^2^ = 0.53) suggests nodal stress as a common mechanism. Corroborating this interpretation with independent data, we show that metabolic ‘cost’ significantly differs along this transdiagnostic/multimodal gradient.

## Abstract (web summary)

Peter Fox & Thomas Vanasse et al. compare atrophy patterns in 40+ brain disorders with healthy task-activation networks across 100+ behavioral domains. Their analyses demonstrate strong spatial correspondence between disease-related structural coalterations and functional networks, an observation that greatly extends the scope of the network-degeneration hypothesis. Further, the metabolic cost of network hubs (nodal stress) is implicated as a common mechanism across a wide range of brain disorders.

## Introduction

Network architecture is a fundamental, multi-scale motif in brain organization, presumably reflecting evolutionary pressure for efficient information-processing. Network properties have been demonstrated across a wide range of spatial scales. The microscale contains connections between individual neurons, while the macroscale comprises distributed systems which encompass direct and indirect connections between more distant brain regions^1^. Progress in neuroscience over the past three decades has been extraordinary, much of which can be attributed to the development of high-resolution, whole-brain imaging methods, and advanced analytic approaches for network discovery. For human neuroscience, functional and structural magnetic resonance imaging (fMRI and sMRI) coupled with data-driven analytic methods applied at the systems level have been particularly impactful^2–4^.

System-level functional networks are defined by their functional connectivity, most often inferred by measuring temporal correlations of neuronal activity^5^. The reliability of functional connectivity as a biological construct has withstood rigorous examination: the functional organization of the brain is dominated by a core architecture shared between individuals (i.e., system-level networks), but stable connectivity features unique to individuals are also present^6^. Substantially less variability in functional brain connectivity is explained by day-to-day variability or even task-state^6^. Thus, functional connectivity is a robust metric to study behavior, cognition, and disease^7^. Fifteen to 20 functional networks are readily identifiable and can account for much of our understanding of brain–behavior ontology^8–10^. These functional circuits are considered to mediate susceptibility to dimensions of psychopathology rather than discrete disorders^11^, making them especially relevant for transdiagnostic investigation^12^.

The network denegation hypothesis (NDH) posits that disease-related structural alteration selectively occurs—and may even spread—within these system-level functional networks^13^. Just like neuronal activity, gray matter structural alteration (atrophy or hypertrophy) has shown to follow network-based principles^14^: structural alteration in one brain area is influenced by alteration in other brain areas^15,16^. This concept is hereon referred to as co-alteration structural connectivity (CA-SC). Previous work has linked four specific neurodegenerative disorders’ atrophy patterns to four corresponding functional circuits^17^, and other work has found shared CA-SC effects in a single functional circuit across six psychiatric diagnoses^18^. In recent years, some studies have even suggested that network-based pathology recapitulating physiology may be a general property of neuropsychiatric disorders that can occur in response to a combination of plausible disease mechanisms^14^. But this fundamental question of NDH, specifically the extent of structural and functional correspondence, remains unclear. Furthermore, toward a refined understanding of NDH, new evidence has even suggested that the variety and unpredictability of diseases that structurally affect a brain area may be associated with regions that are important for cognitive/integrative function^19^. Transdiagnostic disease vulnerability of brain networks has not yet been assessed and compared to functionally based, integrative indexes (i.e., behavioral specialization) in any formal capacity. Confirming this hypothesis would have important implications for further understanding NDH. Functional specialization is proposed to reflect unique metabolic brain characteristics^20^ that may underlie specific NDH mechanisms.

Meta-analytic network analysis, which draws upon decades of human neuroimaging research, has proven to be a powerful way to study brain organization and pathology^21^. Specifically, the BrainMap (www.brainmap.org) database project has involved the manual curation of standardized results (*x*–*y*–*z* brain coordinates) from thousands of whole-brain functional and structural neuroimaging experiments, along with a rich taxonomy of the relevant behavior (i.e., behavioral domain and task paradigm) and disease (i.e., ICD-10 diagnosis) metadata, respectively. In utilizing this dual-modality resource for network analysis and investigating NDH, a plethora of analytic methods are available. Independent component analysis (ICA), which requires perhaps the least assumptions of neuroimaging data as opposed to other analytic models^2,22^, can be applied to meta-analytic data from BrainMap as has been done previously^10,23^. ICA is a multivariate method that identifies a specified number of spatial networks by linearly unmixing whole-brain data into maximally independent sources^24^.

In the first part of this study, we test NDH’s broad-based proposition that network-based structural pathology adheres to the brain’s functional architecture when considering many neuropsychiatric disorders. We test this hypothesis in a data-driven manner by spatially comparing 20 transdiagnostic CA-SC networks to 20 task-activation functional connectivity (TA-FC) ICA networks; each network set was separately generated from their respective BrainMap modality (structure vs. function). We also examine higher model orders (*d* = 45, 70) to assess generalizability. In the second part of this work, utilizing a network-normalized entropy metric derived from metadata loadings (which captures both the diversity and non-specificity of metadata associations), we test the hypothesis that brain networks that are highly behavior entropic are also highly disease entropic. To further investigate the specific NDH mechanistic prediction of nodal stress (NS), we utilize a separate dataset^25^ that reported regional differences in the brain’s metabolic attributes among healthy individuals, and we test whether those markers associate with disease and behavior entropy.

## Results

### Network discovery

Two general characteristics of the CA-SC and TA-FC networks identified at *d* = 20 using ICA were observed. First, no singular (one-to-one) disease-to-network or behavior-to-network matching was clearly evident based on the disease and behavior network loadings. Forty-three distinct disorders were included for structural analysis, and 56 distinct behavior categories were included in the functional analysis. One CA-SC network (medial visual) was unique in that no disease category associated with it above a 75th percentile threshold. None of the TA-FC components demonstrated this sparsity. Second, neurological disorders (ICD G codes) showed stronger CA-SC component associations compared to psychiatric disorders (ICD F codes) in a non-parametric Wilcoxon rank-sum test (*p* = 0.01) of all component-metadata loadings.

### Cross-modality spatial correspondence

**Fig. 1:**
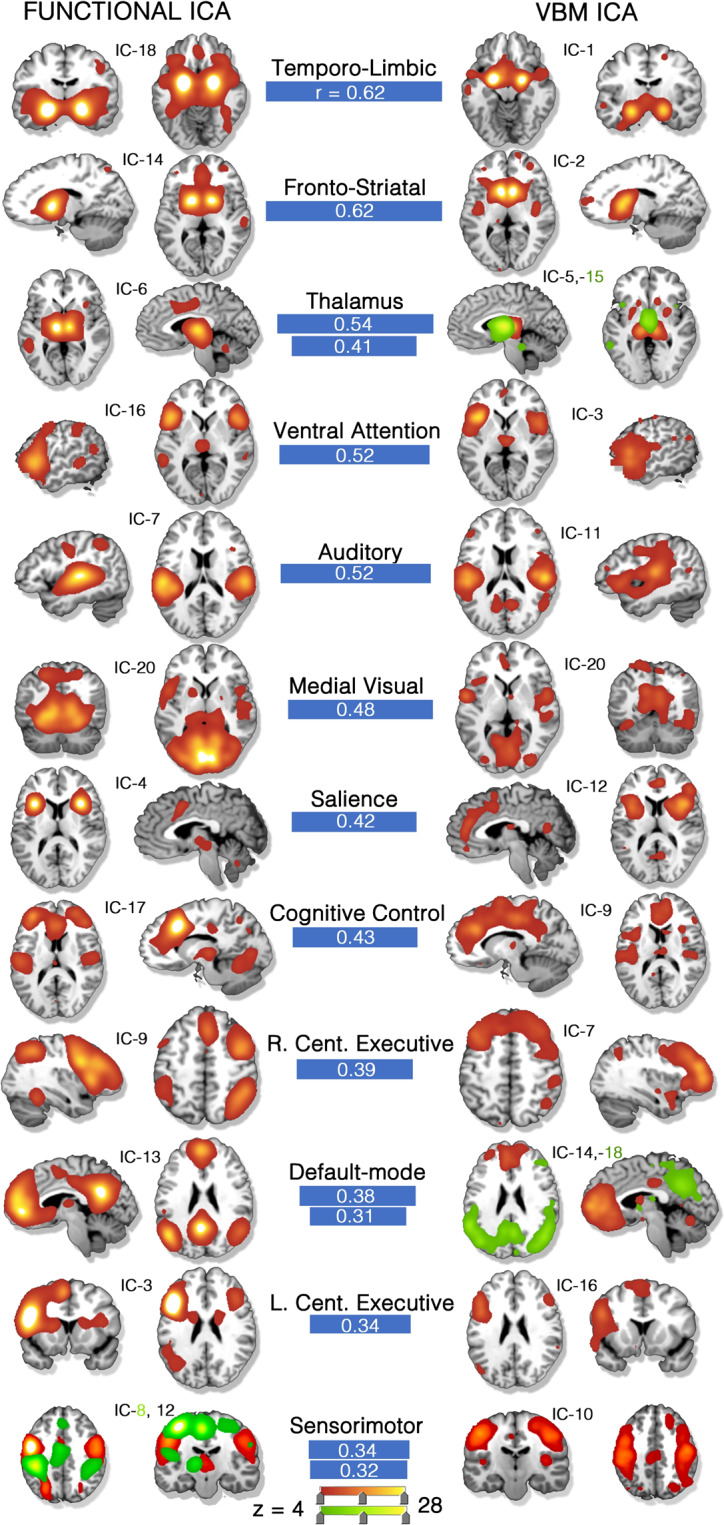
Co-alteration and task-activation network correspondence (*d* = 20). (Left) Thirteen task-activation (TA) networks derived from the BrainMap-TA sector (*n* = 7865 task-activation experiments among healthy subjects). (Right) Fourteen structural co-alteration networks derived from the BrainMap voxel-based morphometry sector (*n* = 2002 experiments across *n* > 40 diseases). Clear network matches across datasets are shown by blue connecting bars, the width of which are proportional to the whole-brain, spatial correlation coefficient. Some networks matched to two, separate opposite-modality networks (red and green). Color scale is shown at the bottom of the image. Independent component (IC) numbers are ordered by explained variance within the respective dataset. All component matches are at or below a significance threshold of *p* = 0.01, family-wise error (FWE) rate corrected. Source data are provided in Supplementary Data 1.

**Fig. 2:**
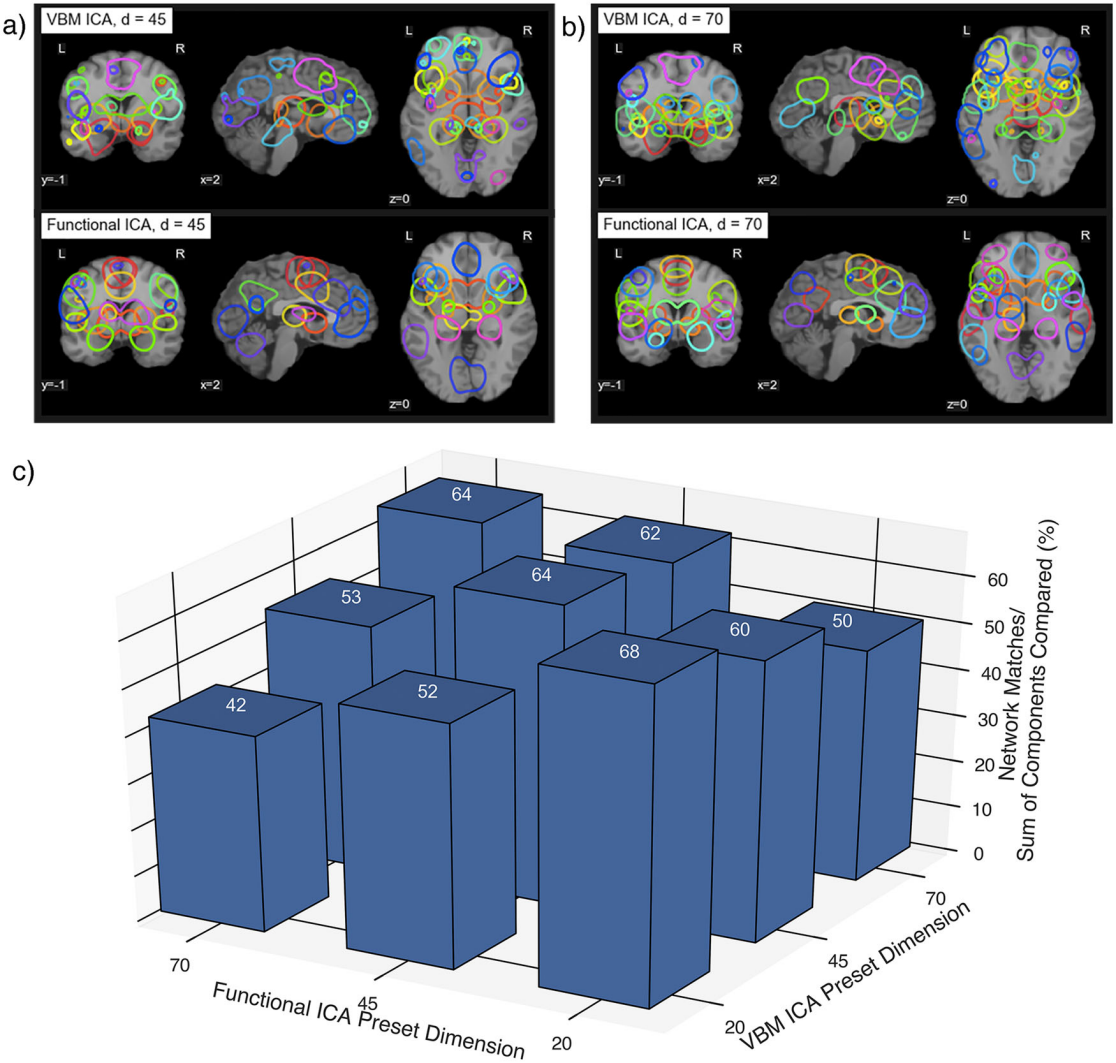
Higher model order comparisons. Components that matched at higher dimensionalities of **a** 45 and **b** 70. **c** Percentage of network matches applying the same correlation threshold across *n* = 9 separate combinations of dimensionalities (20/45/70). Source data are provided in Supplementary Data 1.

Fourteen of 20 CA-SC networks and 13 of 20 TA-FC networks showed clear cross-modal spatial correspondence, and are featured in Fig. 1. These matches met a spatial correlation at *r* ≥ 0.31, corresponding to a family-wise error (FWE) corrected *p* < 0.01. In the post-hoc dimensionality analysis, network correspondence was strongest at *d* = 20 vs. 20 at 68% [14 + 13 matches/(20 + 20) total networks], but remained strong among higher model orders (64%). Percent matching was calculated according to the sum of all unique structural and functional components that had a cross-modality match (*r* ≥ 0.31) divided by the sum of total networks in any specific cross-modality comparison of sets (see Fig. 2). The strongest network correspondence was evident when dimensionality was matched between modalities (i.e., 20/20, 45/45, 70/70) as opposed to off-diagonal comparisons (e.g., 20/45). The upper diagonal, where higher VBM dimensions were compared to lower functional dimensions, showed more matches (mean 57.3%) compared to the lower diagonal (mean 49%).

### Informational comparison

**Fig. 3:**
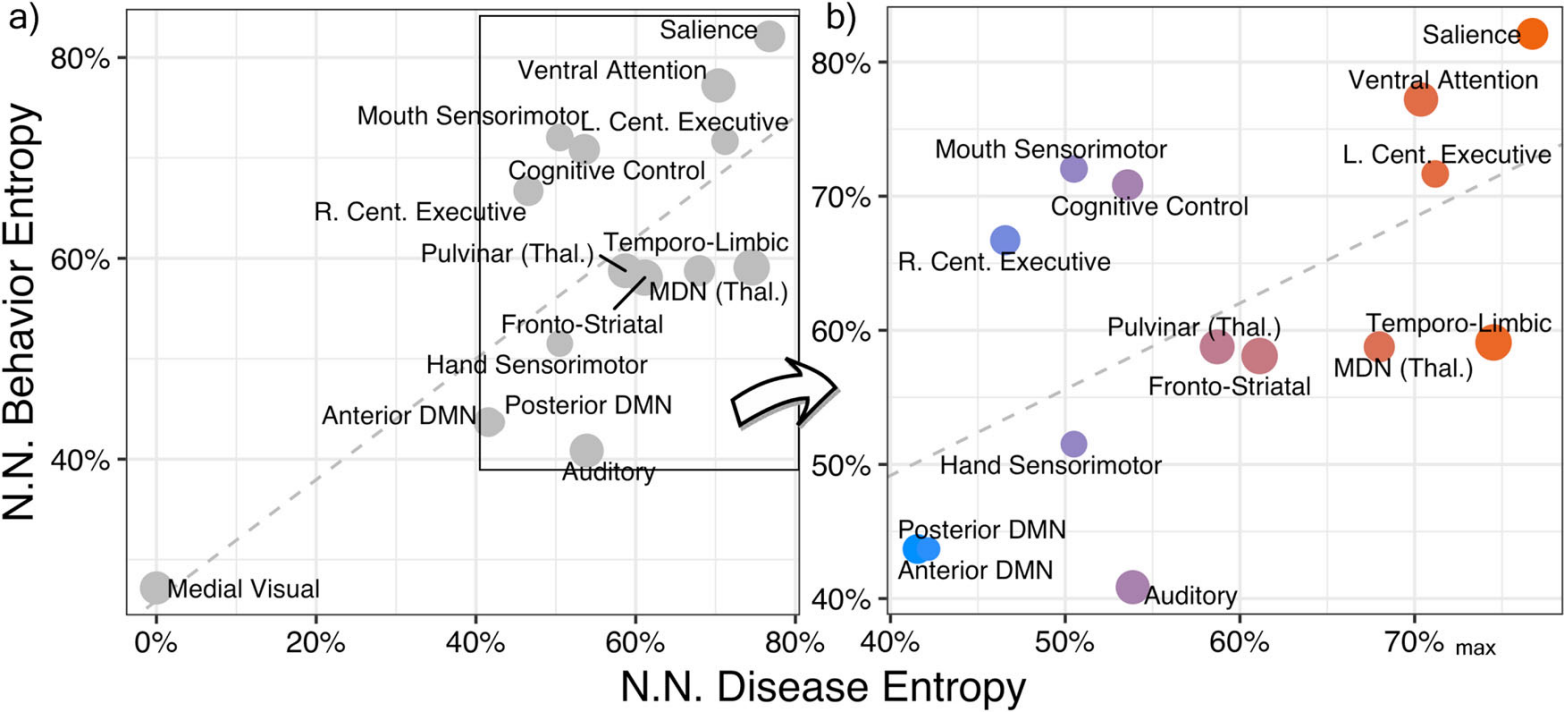
Matched network disease and behavior entropy comparison. The informational content of each network in terms of percent maximum of network normalized behavior and disease entropy. High disease entropy corresponds to a network that is associated with a higher variety of diseases [from *n* = 43 International Classification of Disease (10th version) diagnostic categories] and is non-specific to one or few diseases. High behavior entropy corresponds to a functional network associated with a high variety of Behaviors (from *n* = 56 BrainMap behavior domains) and is also not specialized. Only those *n* = 15 networks that are significantly matched between co-alteration and task-activation ICA are displayed; the size of data points is proportional to their spatial correlation (0.31 ≤ *r* ≤ 0.62). The linear model **a** had the following parameters: *β* = 0.60; *p* = 0.0006; df = 13; Adjusted *R*^2^ = 0.53. Panel **b** excludes the medial visual network from view, where color corresponds to the fitted values of the model. NN network normalized, MDN medial dorsal nucleus, DMN default-mode network, Thal. thalamus, L./R. left/right, Cent. central. Source data are provided in Supplementary Data 1.

Among matched networks, a linear regression was indeed found to be significant between disease (independent variable) and behavior network-normalized entropy among all matched networks [*β* = 0.60 (std. error: 0.147); right-tailed *p* = 0.0006; df = 13] (Fig. 3). Higher-order, supra-modal, associative networks tended to rank high in both behavior and disease entropy. The salience network, for example, ranked highest on both disease and behavior entropy. Similarly, the left central executive and ventral attentional networks were highly disease and behavior entropic. Subcortical nuclei and immediate connections (e.g., pulvinar, medial dorsal nucleus, and corpus striatum), often described as performing relay functions, were intermediate in both behavior and by disease-diversity ranking. Lower-order, unimodal, perceptual, and motor networks (e.g., medial visual, hand sensorimotor, and auditory) were behaviorally sparse and had minimal disease diversity. Most networks fell within the range of 40–80% of the maximum possible entropy value. The medial visual network was an outlier, explaining the least amount of variance within both functional and structural datasets (ranked 20th).

**Fig. 4:**
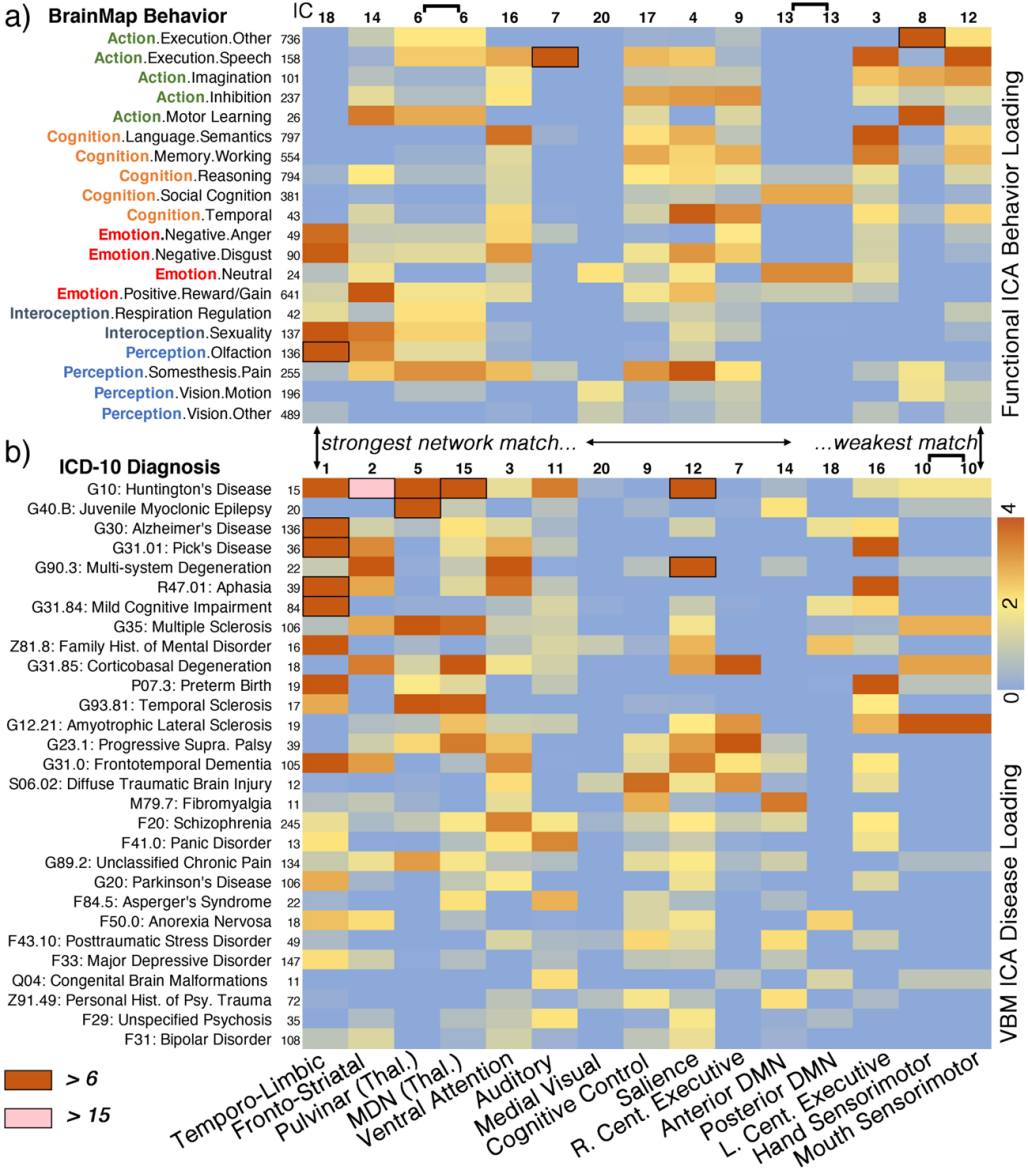
Matched network metadata loadings. **a** Twenty (of *n* = 59) selected behavior domain loadings of task-activation functional networks. **b** Twenty-nine (of *n* = 43) selected disease loadings of co-alteration networks. One column, spanning panels **a** and **b**, corresponds to a network match. Stronger to weaker spatial correspondence is ordered from left to right. Metadata loadings are scaled by median absolute deviation about zero (see the “Methods” section). Metadata experiment volume within the database at the time of analysis are displayed to the right of the metadata label. Cell borders/shading specify more extreme loadings above 6 and 15. Source data are provided in Supplementary Data 1.

Some disease categories had more diffuse loadings across networks than others. G10: Huntington’s disease affected nine matched networks, with loadings greater than the 75th percentile, and was followed by G31.0: Frontotemporal Dementia and F20: Schizophrenia with seven affected networks. G31.85: Corticobasal Degeneration and G23.1: Progressive Supranuclear Palsy each were associated with six networks above the threshold. On the other end of the spectrum, diseases including G40.B: Juvenile Myoclonic Epilepsy, F84.5: Asperger’s Syndrome, F29: Unspecified Psychosis, F31: Bipolar Disorder, and F33: Major Depressive Disorder each had two loadings or less above the 75th percentile among matched networks. The median TA-FC network association across all diseases in Fig. 4 was 4 (diseases per network).

### Metabolic differences

To corroborate our interpretation that behaviorally entropic functional and disease entropic structural brain hubs are indeed associated with increased metabolic cost (an NDH prediction known as NS^13^), we regressed our entropy measures to a group-level, voxelwise metabolic statistical map previously acquired and shared by Shokri-Kojori et al. ^25^. They used positron emission tomography (PET) and fMRI data recorded from 28 human subjects. An energetic supply factor was derived from PET data and a demand factor from fMRI. These factors were then transformed with sinusoidal transformation to provide two new factors: cost (which corresponds roughly to anti-correlation or independence between supply and demand) and power (which corresponds roughly to correlation or dependence between supply and demand).

**Fig. 5:**
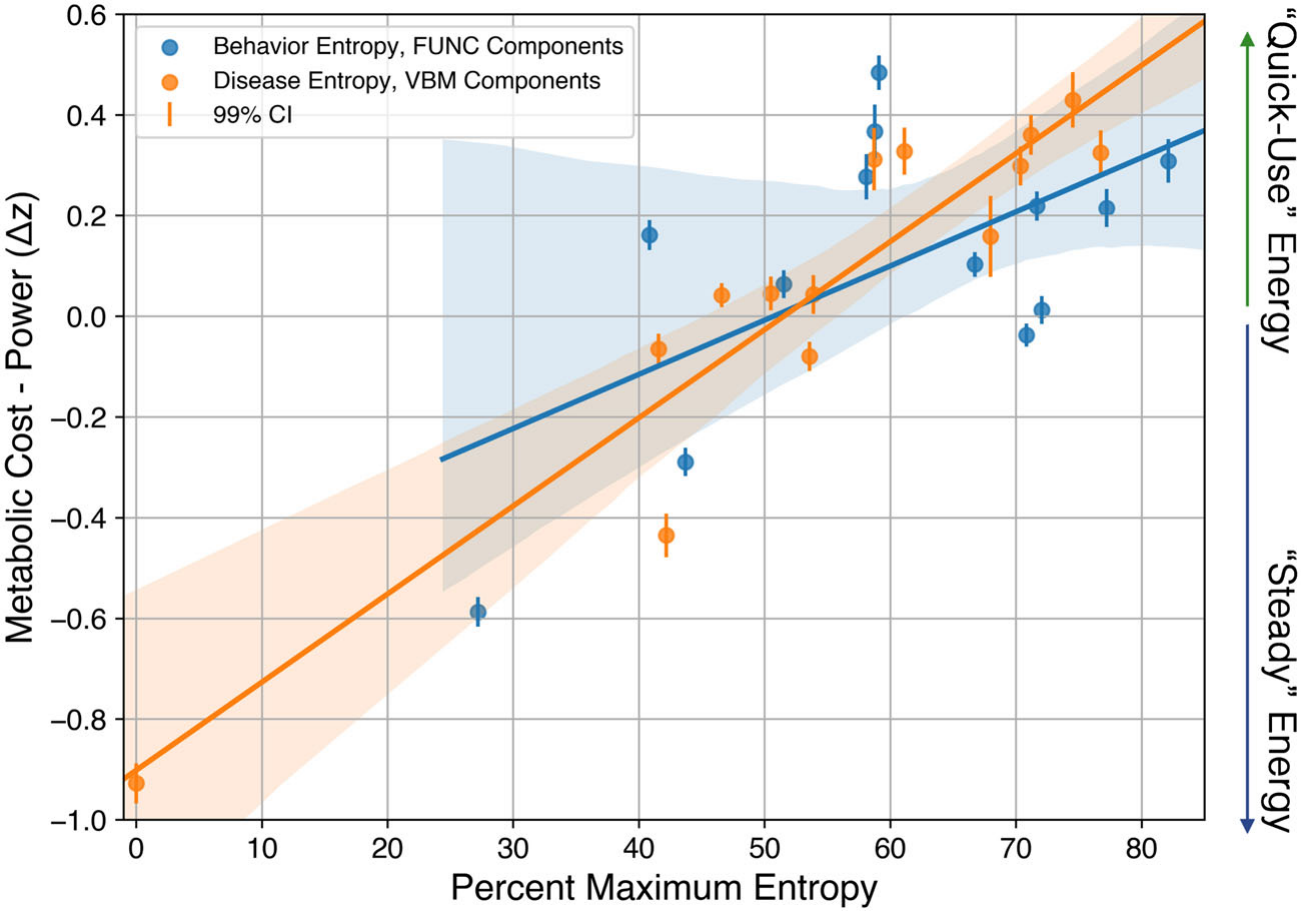
Metabolic brain attributes vs. disease/behavior entropy. Using a published dataset capturing the dynamics of metabolic supply mismatching energetic demand (higher relative cost vs. power) among *n* = 28 healthy subjects, we performed linear regression with *n* = 14 percent maximum disease and *n* = 13 behavior network entropy metrics as separate independent variables (matched VBM and functional components, colored orange and blue, respectively). Both regressions were found to be significant after correcting for multiple tests, *p* = 7e−7 and *p* = 0.03, for disease–structural and behavior–functional network data, respectively. Source data are provided in Supplementary Data 1.

To capture variation in both dimensions, which the authors proposed to represent two separate metabolic pathways (fast aerobic glycolysis vs. steady oxidative metabolism), the difference between cost and power maps was applied here. This difference (cost–power) significantly associated with both disease [*β* = 0.017 (std. error = 0.0018), intercept = −0.90, *R*^2^ = 0.88, Bonferroni-corrected right-tailed *p* = 7.6e−07] and behavior [*β* = 0.011 (std. error = 0.0043), intercept = −0.54, *R*^2^ = 0.35, Bonferroni-corrected right-tailed *p* = 0.03] entropy (Fig. 5).

### Matched network behavioral/pathological profiling

Loading matrices for each matched network are provided in Fig. 4. Each network match is reported below, paragraph-by-paragraph, in order of higher to lower magnitude of correspondence, i.e., spatial correlation coefficient.

The temporo-limbic functional and co-alteration networks both included the bilateral amygdala, anterior cingulate (BA 24/32), and left lateral prefrontal cortex (BA 9/46). The temporo-limbic co-alteration network was loaded on by many diseases including G30: Alzheimer’s disease (9.3–median absolute deviations from zero; see the “Methods” section), G31.84: Mild Cognitive Impairment (6.2), G31:01 Pick’s Disease (6.8), R47.01: Aphasia (6.3), and G31.0: Frontotemporal Dementia (4.0) (Fig. 4). This functional network was loaded on most by Perception.Olfaction (7.8) and Emotion.Negative.Disgust (3.9).

The fronto-striatal functional and co-alteration networks shared anatomy in the bilateral caudate and middle frontal gyri. The co-alteration component was loaded on by G10: Huntington’s Disease (21.7) most, the strongest loading by any disease on any network by a factor of 2. It was also loaded on by G31.85: Corticobasal Degeneration (3.5) and F50.0: Anorexia Nervosa (2.2). Behaviors including Action.Motor Learning (3.5) and Emotion.Positive.Reward (4.9) highly loaded on the fronto-striatal functional network. Other neurodegenerative syndromes that loaded on this component including R47.01: Aphasia, G30: Alzheimer’s disease, and G31.0: frontotemporal dementia.

The thalamus functional network corresponded to two separate co-alteration networks: one primarily included the pulvinar thalamus (*r* = 0.54) and the other mostly included the medial dorsal nucleus (*r* = 0.41). The pulvinar thalamus co-alteration network was loaded on by G40.B: Juvenile Myoclonic Epilepsy (9.3) most. The medial dorsal nucleus co-alteration network was loaded on by G31.85: Corticobasal Degeneration (5.6). Both thalamus co-alteration networks were weighted on by G35: multiple sclerosis and G93.81: Temporal Sclerosis. The thalamus functional network was associated with a diverse set of behaviors including Perception.Somesthesis.Pain (3.2), Action.Motor.Learning (2.8), as well as Interoception.Respiration Regulation (2.1).

The ventral attention functional network matched to a co-alteration network that was loaded on by F20.0: Schizophrenia (3.3), G90.3: Multi-system Degeneration (4.1), R47.01: Aphasia (3.6), and G31.0: Frontotemporal Dementia (3.2). The matched functional and co-alteration networks both included the bilateral inferior frontal gyrus and parts of the anterior insula and thalamus. The functional network supported a variety of tasks, and was loaded on by Cognition.Language.Semantics (3.6), Cognition.Temporal (2.2), as well as Emotion.Negative.Disgust (3.1).

The auditory functional network—which included auditory cortices (BAs 41/42/22)—matched to a co-alteration network that was loaded on by F41.0: Panic Disorder (3.3), F84.5: Asperger’s Syndrome (2.7), and F29: Unspecified Psychosis (2.1). This functional network was uniquely associated with Action.Execution.Speech (6.2).

The matched functional and structural salience networks primarily included the bilateral anterior insula and dorsal anterior cingulate. This network was loaded on by many neurological and psychiatric diseases including G31.0: Frontotemporal Dementia (3.5), G89.2: Unclassified Chronic Pain (1.9), F20: Schizophrenia (2.0), and even Z81.8: Family History of Mental Disorder (2.7). The salience functional network was loaded on most by behaviors including Perception.Somesethesis.Pain (4.1) and Cognition.Temporal (3.9)—the mental faculty associated with the system of sequential relations that any event has to any other as past, present, or future.

The cognitive control network included the anterior cingulate, dorsolateral prefrontal cortex, and posterior insula. The cognitive control co-alteration network was associated with S06.02: diffuse traumatic brain injury (3.7) and F43.10: Posttraumatic Stress Disorder (2.2). Behaviors most associated with this functional network included Perception.Somesthesis.Pain (3.2) and Cognition.Memory.Working (2.9).

The right central executive (i.e., fronto-parietal) functional network was strongly associated with cognitive tasks including Action.Inhibition (3.2) and Cognition.Memory.Working (2.9). This co-alteration network did include bilateral aspects of the middle frontal gyri, and was robustly loaded on by tauopathies including G31.85: Corticobasal Degeneration (4.6) and G23.1: Progressive Supranuclear Palsy (4.0).

The default-mode functional network was matched to separate anterior and posterior co-alteration networks, which were loaded on by disparate diseases. The anterior default-mode co-alteration network was loaded on by Z91.49: Personal History of Psychological Trauma (2.2) and M79.7: Fibromyalgia (3.5), while the posterior aspect was loaded on by G30: Alzheimer’s Disease (1.6), G31.84: Mild Cognitive Impairment (1.6), and Z81.8: Family History of Mental Disorder (2.5). The associated behaviors with the default-mode network included Cognition.Social Cognition (2.9) and Emotion.Neutral (3.3).

The left central executive (i.e., fronto-parietal) network was strongly affiliated with G31.01: Pick’s Disease (4.1), R47.01: Aphasia (4.6) and P07.3: Preterm Birth (4.8). This left-lateralized functional network was associated with Cognition.Language.Semantics (5.7), Action.Execution.Speech (3.9), and Cognition.Memory.Working (3.5).

The sensorimotor co-alteration network—which contained the paracentral lobule (BA 6) and aspects of the postcentral gyrus (BAs 4/3)—was selectively loaded on by G12.21: Amyotrophic Lateral Sclerosis (4.2) and G35: Multiple Sclerosis (2.7). Two functional connectivity networks, (1) hand and (2) mouth sensorimotor, were significantly associated with this co-alteration network. The hand sensorimotor (IC-8) was strongly loaded on by Action.Execution.Other (7.3), Action.Motor.Learning (4.5), and Action.Imagination (2.9) Behavior Domains. The mouth sensorimotor network was loaded on most by Action.Execution.Speech (5.7).

Finally, the medial visual co-alteration network’s highest disease loading was S06.02: diffuse traumatic brain injury at 1.14, but this was still not above the 75th percentile threshold, and thus did not contribute toward disease diversity. The functional network predictably weighted most on Perception.Vision.Motion (1.73).

The functional networks that did not match to a co-alteration network included a dorsal attention network, a somesthesis network (containing posterior insula, inferior parietal lobule, and cingulate), lateral visual (V2) and visual association (V3) networks, two visuomotor coordination networks, and a cerebellum network. Five co-alteration networks that did not have a TA-FC network match included a posterior insula/posterior cingulate network (IC-13), left-lateralized and right-lateralized hippocampus networks (IC-6, 4), a bilateral posterior hippocampus network (IC-17), and an inferior temporal lobe network (IC-8).

## Discussion

This work synthesized and compared the healthy task-activation and disease morphology human neuroimaging literatures at large scale. The main impact of this effort is twofold: (a) we demonstrate that TA-FC architecture comprehensively associates with disease-related structural co-alteration, unequivocally affirming the network degeneration hypothesis (NDH) as a broad-based phenomenon; and, (b) by virtue of mass comparison of structure and function, etiologic inferences can be made about proposed transdiagnostic mechanisms of action, and even about shared symptomotologies across disorders. Utilizing independent brain metabolism data showing regional differences in energy utilization, we argue that metabolic susceptibility—proposedly linked to NS—prominently contributes to transdiagnostic action.

Since Seeley et al.’s seminal work in 2009^17^, the NDH has shifted in scope to include a few neurodegenerative disorders and networks (e.g., Alzheimer’s disease and the default-mode network; or corticobasal syndrome and the somatomotor network), to now—as we show—be powerfully relevant in understanding a panoply of diseases and CA-SC networks. This study comprehensively demonstrates this network-based phenomena in a data-driven manner, using ICA featured at a low model order. We also show that matching persists, with only a slight decrease in percent matching, along higher network dimensionalities. This proves that structural and functional matching was not an artifact of dimensionality selection. Importantly, correspondence was highest when dimensionalities matched across modalities (see diagonal in Fig. 2), which suggests that CA-SC/TA-FC networks fractionate similarly as model order increases. Messe^26^ demonstrated similar results when comparing the healthy structural/functional connectome via graph theory, in that partition matching between modalities remained constant from low-count to high-count atlas parcellations.

The scope of structural and functional network correspondence is noteworthy here in that 14 CA-SC networks (*d* = 20) matched to a TA-FC network, but also the range of disorders (43) involved in this analysis should be of emphasis. Neurological diseases did indeed have stronger network associations than psychiatric diseases, which reinforces the fact that neurological diseases are more neurodegenerative and severe in comparison to psychiatric disease. Severe aggregation of amyloid-β, tau, α-synuclein, and TDP-43 is found post-mortem in virtually all brains with neurodegenerative disease^27^. But some networks also showed a vast array of psychiatric associations (see salience or temporo-limbic network in Fig. 4). It is thus crucial to discuss transdiagnostic mechanisms that may be driving the observed structural patterns in both neurological and psychiatric diseases.

Toward etiological inference, the second part of our work demonstrated a significant linear association between disease diversity and behavior diversity, which likely reflects the transdiagnostic NDH principle of NS. NS, which leads to network degeneration, is perhaps the most evidenced of the pathophysiological theories on offer by NDH as argued by Cauda et al. ^14^ and others^28^. The concept of NS suggests that functional brain hubs, or areas that are highly connected, are most susceptible to many disease mechanisms. The higher end of the linear gradient shown in Fig. 3 (with associated high behavior and disease diversity) contained the salience, ventral attention, and left central executive networks, whose core anatomy, respectively, contained the dorsal anterior cingulate, bilateral anterior insula, and left middle frontal gyrus. Each of these regions has a strong resting-state participation coefficient, a graph theoretical hub measure that characterizes nodes that are involved in multiple subnetworks of the brain^29^. Further down the graded scale of disease diversity were the thalamus and fronto-striatal networks. The thalamus and aspects of the basal ganglia both contain dense structural connections^28^. Finally, at the lower end of the co-alteration disease entropy spectrum, were the sensorimotor, medial visual, and the anterior/posterior default-mode networks. The sensorimotor network was selectively associated with G12.21: amyotrophic lateral sclerosis and G31.85: corticobasal syndrome. The medial visual network had extremely low disease diversity, and did not associate with one disease past the 75th percentile of loadings among all networks and disorders (thus having 0% disease diversity). While the default-mode network was expectedly behaviorally non-diverse (being task-negative^30^), it is important to note that it contains a highly connected region in the posterior cingulate cortex^31^. Even though the posterior cingulate is highly connected to its local community, it does not have strong inter-modular FC^29^.

Higher energetic costs among brain hubs make them vulnerable to structural alteration according to NS, as any disease process leading to metabolic impairments should selectively damage them^28,32^. To test this prediction, we regressed our network entropy results to an independent dataset that characterized metabolic expense while accounting for neuronal activity. Specifically, Shokri-Kojori et al.^25^ looked at network differences in energy dynamics and metabolic supply of the brain by comparing: (a) the extent that energy utilization exceeds activity (relative cost, rCST); and, (b) the extent of concurrent energy utilization and activity (relative power, rPWR). The relative difference between these two measures (rCST–rPWR) captures both dimensions, and significantly associated with both disease and behavior entropy in this study. Higher rCST, as the authors suggest, “may involve the use of faster (but inefficient) metabolic pathways such as aerobic glycolysis”. Acute changes in metabolism occur in response to neuronal stimulation, and increased energy demand causes a Warburg-like transient dissociation between glycolysis and oxidative phosphorylation (for a review of the brain metabolism literature, see ref. ^33^). Functionally specialized networks are instead proposed to rely on more efficient oxidative metabolism (higher rPWR). For example, the default-mode network and the medial visual network both showed low behavior-entropy and disease-entropy in the present analysis. While these networks are highly metabolically active, their steady energetic characteristics are not considered to be as costly^25^, which would leave them less suspectable to disease mechanisms according to NS. Interestingly, disease entropy explained much more variance (*R*^2^ = 0.88) in metabolic attributes than behavior entropy (*R*^2^ = 0.35).

Metabolic abnormalities among neuropsychiatric disorders have been widely reported. Mitochondrial dysfunction in many neurodegenerative diseases is elicited by genetic alterations, exogenous toxins, or buildup of toxic metabolites^34^. Schizophrenia, bipolar disorder, and major depressive disorder have shown common and distinct markers of energy metabolism dysfunction with in vivo magnetic resonance spectroscopy^35,36^ and proteomic analyses of postmortem brain tissue^37^. Animal models have recently suggested that elevated glycolysis may underlie increases in lactate and pyruvate levels observed across multiple psychiatric disorders^38^. Finally, oxidative stress is thought to be involved in neurodegeneration observed across Alzheimer’s disease, Parkinson’s disease, and Huntington’s disease among other disorders^39^. While we highlight the role of NS here, this does not preclude the possibility that other mechanisms including prion-like transsynaptic spread^40^ or shared genetic susceptibility^41^ also contribute to CA-SC, but we argue to a lesser extent when considering a vast array of disorders.

Some diseases affected a variety of TA-FC networks as opposed to a few. These include G10: Huntington’s disease, G31.0: frontotemporal dementia, F20: Schizophrenia, G31.85: corticobasal degeneration, and G23.1: progressive supranuclear palsy. It is difficult to broadly speculate about this observation beyond the fact that each of these diseases has a multitude of severe symptoms. G10: Huntington’s disease most obviously impairs movement in gait and speech, but it also manifests cognitive problems and psychosis in many patients^42^. Furthermore, as with many other dementias including G31.0: frontotemporal dementia, even olfaction is impaired^43^. Conversely, F33: major depressive disorder was only weakly associated with the temporo-limbic network in this analysis, which is likely a result of its clinical heterogeneity and the overall difficulty of finding a robust neuroimaging signature of this disorder^44,45^. Separately, G40.B: juvenile myoclonic epilepsy strongly and uniquely loaded on the pulvinar thalamus network, suggesting that this disease has a more focal mechanistic action.

Other approaches to identify multivariate associations between two or more distinct neuroimaging modalities include, but are not limited to, Joint ICA^46^ and Linked ICA^47,48^. Both of these methods require multi-modality data from each subject, which is not clearly applicable to the meta-analytic dataset here. Still, this literature provides us some noteworthy insight into how TA-FC and structural co-alteration can be mutually affected by disease. For example, Joint ICA has identified schizophrenia gray matter effects in bilateral parietal/frontal, and right temporal regions to be associated with activations by an auditory oddball stimulus in bilateral temporal regions^46^. Calhoun and Sui extensively reviewed the fusion of structural and functional data applied to schizophrenia, mood disorders, and other psychopathologies in ref. ^49^. These methods offer a promising way forward in uncovering the links between structure and function among many diseases.

One limitation of this work considers that the mean spatial correlation coefficients between the input features (i.e., 12-mm FWHM smoothed coordinate pseudo-activation image per experiment) and the extracted components (i.e., ICA spatial network z-maps) are small in terms of explained variance, and can be difficult to interpret individually (see Supplementary Fig. 2). The spherical assumption of activation or alteration surrounding coordinates is imprecise in comparison to the spatial intricacies of a derived ICA component map with which they are being correlated. This perhaps contributed to the small correlation magnitudes. Expectedly, the number of foci per experiment also played a role in correlation magnitudes. Some higher-foci experiments seemed to activate multiple networks, which likely reduced correlations to a single network. Lower-foci experiments associated with that single network (or subset of a network) had relatively higher correlations. To help the reader intuit the amount of overlap between experiment-level data and extracted components, we have provided a range of high/medium/low experiment-to-network spatial correlations with their corresponding anatomical layouts in Supplementary Fig. 5.

In summary, a broadly based interpretation of the NDH was overwhelmingly confirmed by this comprehensive analysis in that 14/20 of CA-SC each spatially corresponded to a TA-FC network. We found a positive, graded relationship between network-based disease and behavior entropy. Because more behaviorally diverse and non-specialized regions necessarily incorporate hub regions, we interpret this association to reflect the transdiagnostic NS principle. The major metabolic susceptibility to disease inferred here (i.e., NS) could be further addressed in future work by utilizing a database of voxel-based physiology (resting-state metabolism, blood flow)—a term/concept introduced by Gray et al.^44^—and comparing TA, VBM, and voxel-based pathophysiology. We hope that future work can focus on transdiagnostic vulnerability in certain quick-use metabolic pathways, which our data evidently implicate. Finally, researchers can take these multi-dimensional results as a roadmap for more specific investigations since biologically meaningful regions-of-interest can be derived from the component maps shared here^50^.

## Methods

### Data

All data utilized in the analyses reported here were obtained from the BrainMap^®^ database (www.brainmap.org)^51^. BrainMap is an on-line repository of data gleaned by hand curation from the peer-reviewed, English language literature reporting voxel-wise, whole-brain neuroimaging studies, and tabulating significant effects using 3-D spatial coordinates referable to established brain atlas spaces. This online database archives tabular reduced data (standardized spatial coordinates) and experimental-design metadata gleaned from group-wise contrasts, with no per-subject data or personal identifiers. These data formats are classified as not human subjects data and are exempt from Institutional Review Board oversight.

### Independent component analysis

Meta-analytic connectivity modeling of coordinate-based data is a well-established method for determining TA-FC at a comprehensive scale^52^. Consistent with previous ICA investigations utilizing BrainMap^9,10,23,53^, peak coordinates were grouped per experiment in the BrainMap-TA database and smoothed using a Gaussian distribution (FWHM = 12-mm) for pseudo-activation images with 2 × 2 × 2-mm resolution in standardized Talairach space. To limit within-group effects as discussed by Turkeltaub et al.^54^, papers that included three or more experiments with redundant *x*–*y*–*z* coordinates were not included for ICA analysis—an ICA preparatory scheme similar to that of Vanasse et al.^55^ in the VBM database. Thus, 7865 experimental contrasts involving healthy human subjects were used as pseudo-time-point input for ICA analysis. Spatial ICA was applied to the dataset using multivariate exploratory linear optimized decomposition into independent components (MELODIC)^3^ in FMRIB Software Library (FSL)^56^. The CA-SC networks were generated from 2002 VBM experiments representing data from >40 brain disorders^55^. Of note, one of these CA-SC components (VBM IC-19) was anatomically diffuse among white matter, and was considered artifactual. Therefore, this component was not considered in the present analysis.

The pre-set dimensionality of 20 for functional ICA was chosen for multiple reasons. First, 20 components has been empirically shown to provide one of the most informative decompositions of the BrainMap in an analysis of 20 different model orders by Ray et al. ^9^, and in a resting-state dataset from 1414 volunteers collected independently at 35 international centers^8^. Second, 20 components match the dimensionality of that chosen in the co-alteration network analysis^55^, of which the components were planned to be spatially compared.

### Spatial correspondence

To measure correspondence between VBM-ICA and Functional-ICA components, voxel-wise spatial correlation (Pearson’s product–moment) was applied across all pair-wise combinations of components (20 × 19 = 380). Each component match was statistically significant at *p* = 0.01, FWE corrected for multiple comparisons. Our statistical inference approach was based on a FWE method^57^ utilizing simulated Gaussian noise images with spatial smoothness resembling that of the independent components—as employed by Smith et al.^58^. This procedure is detailed in the “Spatial correlation statistical inference” section of the Supplementary Methods, and Supplementary Fig. 1 in the Supplementary Figures.

In the dimensionality analysis, we applied two higher model orders of ICA: 45 and 70. We chose these dimensions because a previous investigation of BrainMap^9^ found both 20 and 70 to be the most informative decompositions, and 45 was in between both. We apply the same correlation threshold empirically derived to compare correspondence across model orders. We counted matches greater than this threshold among all combinations of dimensionalities (*d* = 20, 45, 70), and then normalize that count by dividing by the sum of dimensionality of both component sets (i.e. 20, 45, or 70).

### Metadata–component association

The behavior domain taxonomic framework utilized in this report was created by Fox et al.^59^ and includes 8 action subcategories (e.g., motor learning), 16 cognition subcategories, 15 emotion subcategories, 8 interoception categories, and 9 perception subcategories. The disease category framework followed that of the 10th version of the International Classification of Disease codes (ICD-10) maintained by the World Health Organization^60^.

The association of categorized behaviors and diseases to specific independent components (i.e., z-score spatial maps) involved a two-step approach: (a) the average spatial correlation of each ICA-inputted experiment image within a metadata category (e.g., G30: Alzheimer’s disease) to each component (e.g., default-mode network; masked with positive z-scores only) was calculated (behaviors per functional ICA components; diseases per VBM ICA components)^55^; and, (b) then—for visualization purposes in Fig. 4—loading parameters from selected metadata categories were scaled to interpret their strength. More information regarding this procedure is provided in the “Component weights and scaling” (per behavior/disease category) section in the Supplementary Methods, and in Supplementary Fig. 2. We further tested the consistency of metadata loadings across modalities among matched components, which is detailed in the “Metadata matching across modalities” section of the Supplementary Methods, and Supplementary Fig. 4.

### Disease and behavioral entropy

To quantitatively compare the informational content of each co-alteration and functional network, we extended the voxel-wise entropy concept introduced by Cauda et al.^19^ in the BrainMap-VBM database and Anderson et al.^61^ in the BrainMap-TA database. Entropy captures the predictability of a probability distribution: if fewer states of a system are more likely than others, entropy is lower; if more states of a system are equally likely, entropy is higher. We considered the behavior and disease component loading matrices separately, and included all Behavioral Domains and Diseases with 10 or more experiments at the time of analysis (*N* = 56 behaviors; *N* = 43 ICD-10 diseases).

First, all loadings below the 75th percentile were zeroed (max {*P*_75_,*x*}) because negative values can be effectively interpreted to have null loading [anti-correlation cannot be inferred from the unsigned meta-analytic data employed here^10^], and we did not want relatively weak positive loadings to contribute to our entropy measure—i.e., we zeroed those likely noise loadings only slightly above zero. To assess our chosen percentile threshold more thoroughly, we also provide the results using a 70th and 80th percentile threshold in Supplementary Fig. 3. After this thresholding, the probability of a component (*j*) being in a certain disorder or behavior (*i*) state was inferred via normalization by dividing a component’s sum total loading of all *N* = 43 diseases or *N* = 56 behaviors. Then we calculated the alteration and behavior entropy of that co-alteration component, *j*^62^:

1 $${\mathrm{{Network}}} - {\mathrm{{Normalized}}}\left( {{\mathrm{{IC}}}_j} \right){\mathrm{{Entropy}}} = \mathop {\sum }\limits_{i = 1}^N p_{i,j}{\mathrm{{ln}}}\left( {p_{i,j}} \right)$$

To better interpret the entropy number (whose units are nats), we calculated the percent maximum entropy based on the highest possible value of from a discretized distribution: a uniform probability density function^63^.

2 $${\mathrm{\% }}{\mathrm{{Max}}}\,{\mathrm{{Entropy}}} = \frac{{{\mathrm{{IC}}}_j\,{\mathrm{{Entropy}}}}}{{{\mathrm{{ln}}}(N)}}$$

Finally, behavior and disease entropy loadings (% max) among matched networks were displayed in a scatter plot. A linear regression of network-normalized disease entropy (independent variable) and network-normalized behavior entropy (dependent variable) was tested.

### Metabolic cost and power

To perform comparative analyses with metabolic brain attributes, a data request was made to Shokri-Kojori and colleagues in regard to their recently published paper in *Nature Communications*^25^. They measured both cerebral metabolic rate of glucose (CMRglc, indexed by ^18^F-flurodeoxyglucose; fluorodeoxyglucose-PET (FDG-PET)) and synchronous fluctuations in the blood oxygenation level dependent (BOLD; measured by fMRI and indexed by local functional connectivity density: lFCD) among *n* = 28 healthy individuals. They then computed voxelwise measures of rPWR and rCST by characterizing lFCD-CMRglc dynamics (indexing components of neuronal activity demand and metabolic supply) to classify the brain into major segments based on rPWR and rCST. Only group-level voxelwise rPWR and rCST statistical maps were acquired and used in the present analysis. Specifically, the rCST and rPWR were subtracted to assess the relative differences in brain areas between both measures. Mean cost–mean power values per network were assessed by thresholding components at *z* > 5.

### Statistics and reproducibility

Raw experimental *x*–*y*–z coordinate data and accompanying metadata was accessed from the BrainMap database (http://brainmap.org) with a collaborative use license agreement (http://brainmap.org/collaborations.html); this data included 2002 and 7865 VBM and functional experimental contrasts, respectively. Most computations and visualizations were performed in the Python scientific computing engine. Software packages including Mango (http://ric.uthscsa.edu/mango/), Nilearn (version 0.6.0b)^64^, Nibabel (version 2.5.1)^65^, and Nipype (version 1.4.2)^66^ were heavily utilized for neuroimaging statistics and visualization.

### Reporting summary

Further information on research design is available in the Nature Research Reporting Summary linked to this article.

## Supplementary information

Supplementary Information

Description of Additional Supplementary Files

Supplementary Data 1

Reporting Summary

## Data Availability

All data utilized in the analyses reported here are available on-line from the BrainMap^®^ database (www.brainmap.org). All ICA-computed component maps and average metadata-component loading correlations can be downloaded at BrainMap’s affiliated publication repository (http://brainmap.org/pubs/). Intermediate, per-experiment data formats used in ICA computation (modeled atrophy maps and modeled activation maps) are available upon reasonable request via execution of a data-use agreement and with investigator support. Source data for Figs. 1–5 are available in Supplementary Data 1.

## Code availability

Independent component analyses were performed using the multivariate exploratory linear optimized decomposition into independent components (MELODIC) function of the FMRIB Software Library (FSL) analytic package. Statistical analysis code used to generate figures is shared at TJV’s github page (https://github.com/tvanasse/brainmap_structure_function_2020). Software packages including Nilearn (version 0.6.0b), Nibabel (version 2.5.1), and Nipype (version 1.4.2) were also utilized.
