# Supplementary material for: Brain pathology recapitulates physiology: A network meta-analysis
Source: Commun Biol. 2021 Mar 8;4:301. doi: 10.1038/s42003-021-01832-9 (PMC7940476; doi:10.1038/s42003-021-01832-9)
Supplement: Supplementary file 1 — Supplementary Information [file 42003_2021_1832_MOESM1_ESM.pdf]

Supplementary Materials

Brain pathology recapitulates physiology: A network meta-analysis

Thomas J. Vanasse[†,1], Peter T. Fox[†,2,3,4*], P. Mickle Fox[2], Franco Cauda[5], Tommaso Costa[5], Stephen M. Smith[6], Simon B. Eickhoff[7,8], Jack L. Lancaster[2,3]

[1]Department of Psychiatry, University of Wisconsin-Madison, Madison, Wisconsin, USA

[2]Research Imaging Institute, University of Texas Health Science Center at San Antonio, San Antonio, Texas, USA

[3]Department of Radiology, University of Texas Health Science Center at San Antonio, San Antonio, Texas, USA

[4]South Texas Veterans Health Care System, San Antonio, Texas, USA

[5]FocusLab and GCS-fMRI, University of Turin and Koelliker Hospital, Turin, Italy

[6]Wellcome Centre for Integrative Neuroimaging (WIN FMRIB), Oxford University, Oxford, United Kingdom

[7]Institute of Systems Neuroscience, Medical Faculty, Heinrich Heine University Düsseldorf, Düsseldorf, Germany

[8]Institute of Neuroscience and Medicine, Brain & Behaviour (INM-7), Research Centre Jülich, Jülich, Germany

[†]These authors contributed to the work equally.

*Corresponding Author: Peter T. Fox

Email: fox@uthscsa.edu

Supplementary Methods

Spatial Correlation Statistical Inference. To identify the statistical significance of spatial correspondence between any VBM and FUNCTIONAL ICA component pair, we employed a family-wise error (FWE) strategy based on that of Smith et al.[1]. To create an empirical null distribution, 1,000 iterations of noise simulations were performed. For each iteration, we used our 19 non-artifactual VBM ICA images (real data) and ran spatial correlations to 20 Gaussian noise images that were smoothed with a Gaussian filter matching the FWHM estimate (using fsl_smoothest function) for the functional component images. This procedure created a correlation matrix of 19x20. The maximum correlation of the entire correlation matrix was logged for each iteration. This approach of using the maximum of the correlation matrix as a correspondence statistic provides family-wise control for multiple comparisons[2,3]. The number of simulated r coefficients greater than some threshold divided by the total number of iterations thus provides a p-value.

Our chosen spatial correlation threshold of r = 0.31 corresponds to p=0.01, family-wise error (FWE) rate corrected. Furthermore, if there was a large discrepancy between two or more components as possible candidates for a match to one component ($\Delta r > 0.20$), then only the stronger association was considered a match because clear fractionation could not be concluded.

Component Weights and Scaling (per Behavior/Disease Category). In Figure 4, metadata categories with relatively high component loadings were selected to showcase those that strongly contributed to an independent component's generation. N = 20 (of 56) Behavior Domains and N = 29 (of 43) ICD-10 Diseases were chosen to display. The metadata association matrices were separated into Behaviors (13/20 TA-FC networks that matched to VBM ICA components), and Diseases (14/20 VBM ICA Components that matched to on TA-FC

component). To facilitate visualization and interpretation of loading scores, we chose a scaling approach using a modification of the median absolute deviation (MAD): the median absolute deviation about zero ($MAD_{\mu_0=0}$). MAD (Equation 1) is an alternative measure of dispersion to the standard deviation. MAD is a robust measure, being less influenced by large deviations in the distribution compared to standard deviation[4].

Equation 1

$$\mathrm{MAD} = \mathrm{median}(|X_i - \mathrm{median}(X)|)$$

Because zero was an absolute null-point in spatial correlation weighting, where there is no metadata association, we used:

Equation 2

$$\mathrm{MAD}_{\mu_0=0} = \mathrm{median}(|X_i - 0|) = \mathrm{median}(|X|)$$

to calculate the dispersion about zero. Accordingly, we scaled the Disease Loading matrix and the Behavior Loading matrix separately:

Equation 3:

$$\text{Disease Loading Score} = \frac{X_i}{\text{Disease Matrix } \mathrm{MAD}_{\mu_0=0}} = \frac{X_i}{0.017}$$

Equation 4:

$$\text{Behavior Loading Score} = \frac{X_i}{\text{Behavior Matrix } \mathrm{MAD}_{\mu_0=0}} = \frac{X_i}{0.020}$$

The probability density plots for each metadata loading matrix with scaling marks are shown in Sup. Figure 2.

To help interpret raw loadings, we have provided experiment-level data and corresponding components in Sup. Figure 5.

Metadata Matching Across Modalities. While spatial matching between networks was evaluated, we also aimed to confirm that Behavior and Disease loadings showed broad overlap across matched networks. To put this broader question in a specific example: does the Behavior category '*Emotion.Reward*' similarly load on the fronto-striatal vbm component as it does to the spatially matched fronto-striatal functional component?

As expected, the correlation coefficient between matched component loading matrices for behaviors (r = 0.76, rho=0.72) and diseases (r = 0.8, rho = 0.67) were high. Partial correlations after adjusting for the match-specific magnitude of correspondence between modalities did not appreciably alter the results (< 3% in correlation magnitude), which suggests that mismatch in loadings were not systematically driven by weaker spatial matches in any major way. As Sup. Figure 4 demonstrates, both graphs show a slight negative bias: the linear fitted plots are slightly below unity. This means that a metadata association with a network from its opposite modality (i.e., Behavior Loading-VBM Network) is slightly lower than its corresponding modality (i.e., Behavior Loading-Functional Network). This is to be expected as the functional and vbm matches are not perfect, but they show "reciprocal" validity.

Supplementary Figures

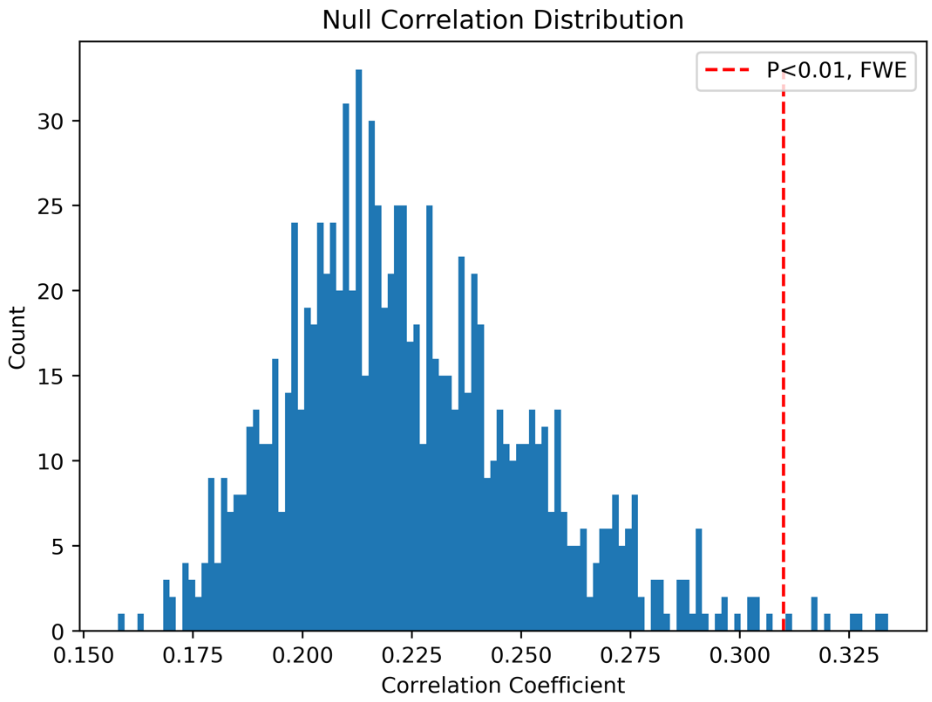

Supplementary Figure 1. In the 1,000 iteration family-wise error rate (FWE) procedure employed here, 20*1,000 noise images were simulated in total to produce a null-distribution.

a)

b)

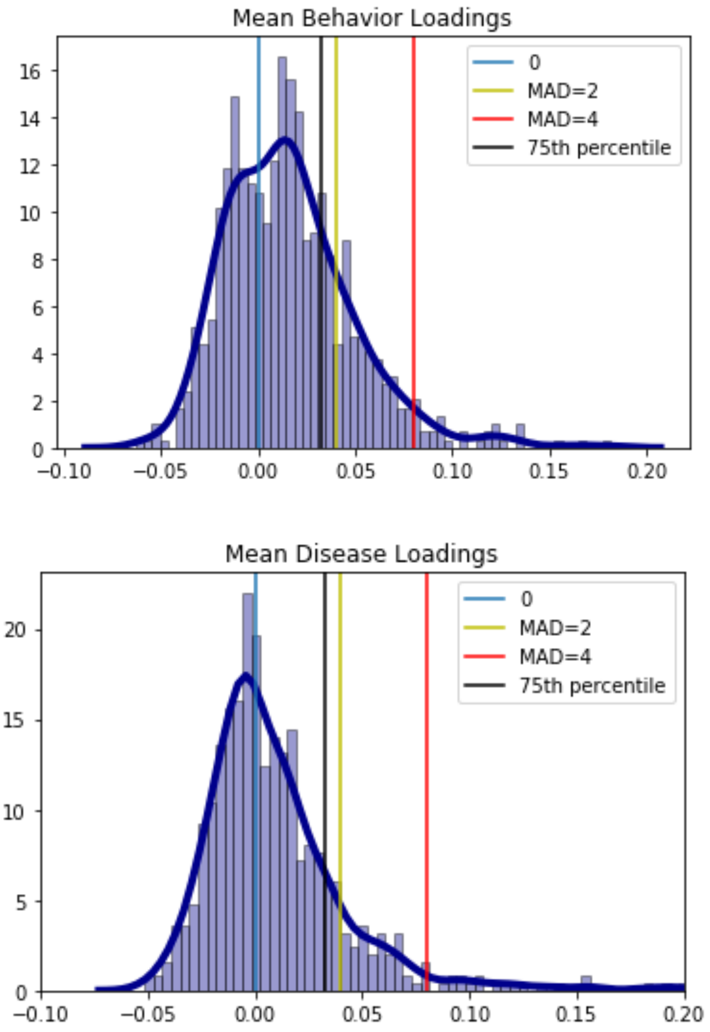

Supplementary Figure 2a-b. Probability density plots for the mean metadata-component association correlations (pre-scaled) derived from all 56*13=728 Behavior-Component mean correlations (a) and 43*13=602 Disease-Component mean correlations (b). The second median absolute deviation from zero is shown in yellow (i.e., scaling score = 2 in Figure 4). The fourth median absolute deviation from zero is red (i.e., scaling score = 4 in Figure 4). The black line corresponds to the 75th percentile, which was chosen for zeroing in our entropy analysis.

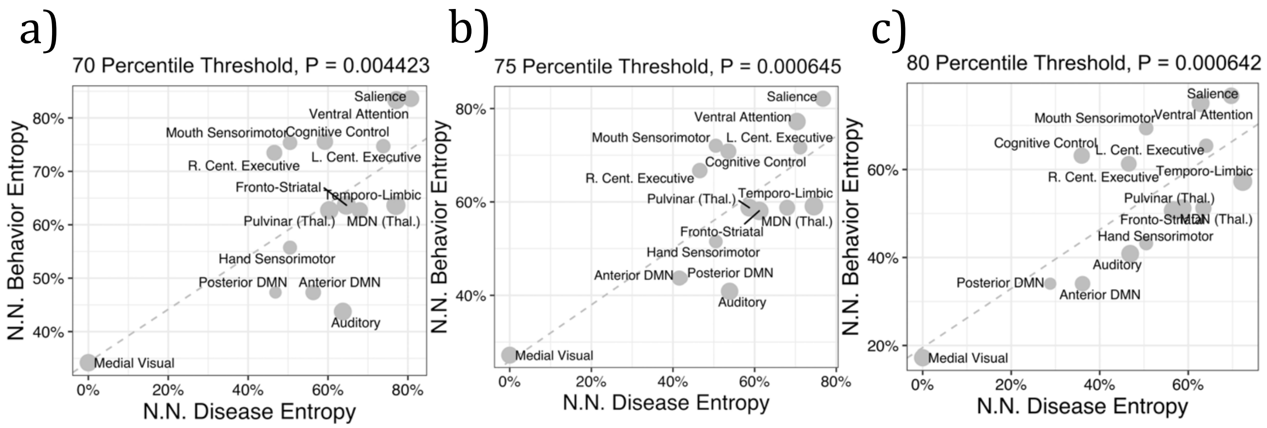

Supplementary Figure 3a-c. Disease Entropy vs. Behavior Entropy linear regression plots with corresponding loading thresholds for our entropy measure. To check the stability of our result, alternate thresholds were chosen 5 percentiles below (A) and above (C) our selected threshold (75th percentile, B). Each regression remains significant after correcting for multiple tests.

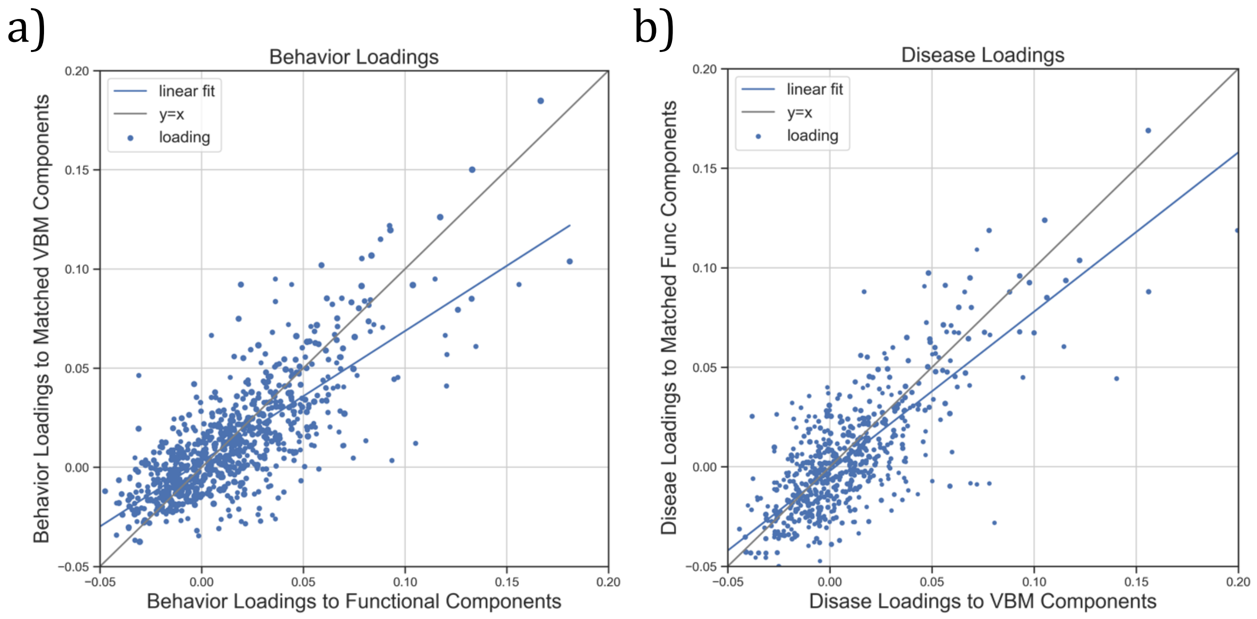

Supplementary Figure 4a-b. Reciprocal loadings across modalities. The grey line represents the unity (y=x) plot, whereas the blue line corresponds to the linear fit to the data. Both graphs demonstrate a slight negative bias in opposite-modality loading (y-axis).

Example A, Experiment 5040011_3 (BrainMapID_Exp)
r = 0.156 (6 foci)
Behavioral Domain(s): *Cognition.Reasoning, Cognition.Attention*

a)

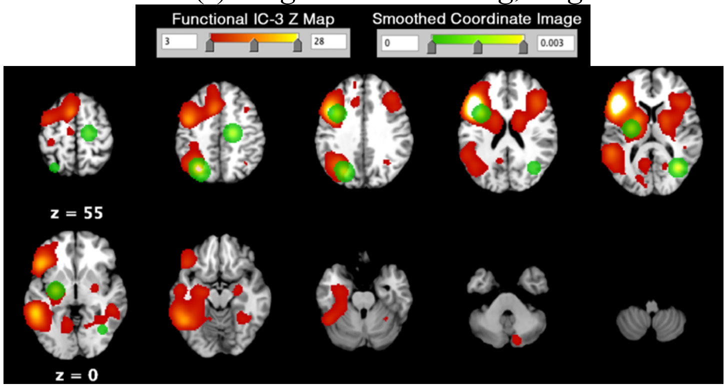

Example B, Experiment 5040007_1
r = 0.0798 (16 total foci)
Behavioral Domain(s): *Perception.Audition*

b)

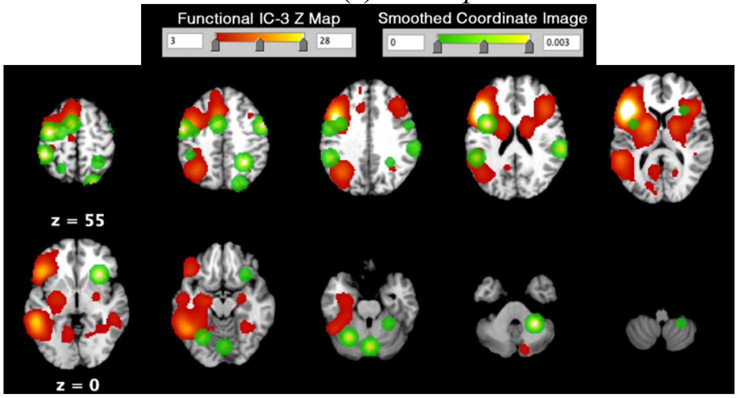

Example C, Experiment 11080075_1
r = 0.0322 (16 total foci)
Behavioral Domain(s): *Cognition.Attention, Emotion.Positive.Reward/Gain*

c)

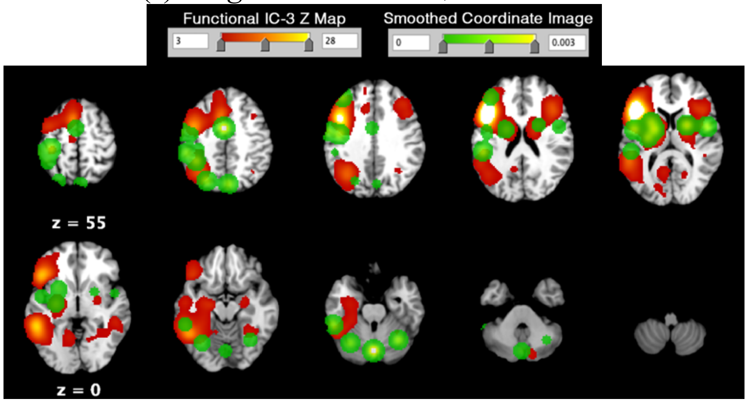

Supplementary Figure 5a-c. Experiment-Level correlations between smoothed three coordinate images (a,b,c) and example ICA component map (Functional IC-3).

Supplementary References

1. Smith, S. *et al.* Structural Variability in the Human Brain Reflects Fine-Grained Functional Architecture at the Population Level. *J. Neurosci.* 39, 6136–6149 (2019).
2. Westfall, P. H. & Young, S. S. *Resampling-Based Multiple Testing: Examples and Methods for p-Value Adjustment*. (Wiley, 1993).
3. Alexander-Bloch, A. *et al.* On testing for spatial correspondence between maps of human brain structure and function. *Neuroimage* (2018). doi:10.1016/j.neuroimage.2018.05.070
4. Leys, C., Ley, C., Klein, O., Bernard, P. & Licata, L. Detecting outliers: Do not use standard deviation around the mean, use absolute deviation around the median. *Journal of Experimental Social Psychology* 49, 764–766 (2013).
