# Supplementary material for: Brain pathology recapitulates physiology: A network meta-analysis
Source: Commun Biol. 2021 Mar 8;4:301. doi: 10.1038/s42003-021-01832-9 (PMC7940476; doi:10.1038/s42003-021-01832-9)
Supplement: Supplementary file 2 — Description of Additional Supplementary Files [file 42003_2021_1832_MOESM2_ESM.pdf]

Description of Additional Supplementary Files

File name: Supplementary Data 1

Description: Data are provided for Figures 1-5.
