# Supplementary material for: Brain pathology recapitulates physiology: A network meta-analysis
Source: Commun Biol. 2021 Mar 8;4:301. doi: 10.1038/s42003-021-01832-9 (PMC7940476; doi:10.1038/s42003-021-01832-9)
Supplement: Supplementary file 4 — Reporting Summary [file 42003_2021_1832_MOESM4_ESM.pdf]

nature research

Corresponding author(s): Peter T. Fox

Last updated by author(s): Feb 4, 2021

Reporting Summary

Nature Research wishes to improve the reproducibility of the work that we publish. This form provides structure for consistency and transparency in reporting. For further information on Nature Research policies, see our Editorial Policies and the Editorial Policy Checklist.

Please do not complete any field with "not applicable" or n/a. Refer to the help text for what text to use if an item is not relevant to your study. For final submission: please carefully check your responses for accuracy; you will not be able to make changes later.

Statistics

For all statistical analyses, confirm that the following items are present in the figure legend, table legend, main text, or Methods section. n/a Confirmed

- The exact sample size (*n*) for each experimental group/condition, given as a discrete number and unit of measurement
- A statement on whether measurements were taken from distinct samples or whether the same sample was measured repeatedly The statistical test(s) used AND whether they are one- or two-sided
  Only common tests should be described solely by name; describe more complex techniques in the Methods section.
- A description of all covariates tested
- A description of any assumptions or corrections, such as tests of normality and adjustment for multiple comparisons
- A full description of the statistical parameters including central tendency (e.g. means) or other basic estimates (e.g. regression coefficient) AND variation (e.g. standard deviation) or associated estimates of uncertainty (e.g. confidence intervals)
- For null hypothesis testing, the test statistic (e.g. *F*, *t*, *r*) with confidence intervals, effect sizes, degrees of freedom and *P* value noted
  Give P values as exact values whenever suitable.
- For Bayesian analysis, information on the choice of priors and Markov chain Monte Carlo settings
- For hierarchical and complex designs, identification of the appropriate level for tests and full reporting of outcomes Estimates of effect sizes (e.g. Cohen's *d*, Pearson's *r*), indicating how they were calculated

Our web collection on statistics for biologists contains articles on many of the points above.

Software and code

Policy information about availability of computer code

Data collection	All data employed in this study were obtained from the BrainMap database (www.brainmap.org), an on-line repository of data gleaned by hand curation from the peer-reviewed, English language literature which reported voxel-wise, whole-brain studies and tabulated significant effects using 3-D coordinates referable to an established brain atlas space (either Talairach or MNI).
Data analysis	Independent component analyses were performed using the MELODIC (multivariate exploratory linear optimized decomposition into independent components) function of the FSL (FMRIB Software Library) analytic package. 5tatistical analysis code used to generate figures is shared at TJV's github page (https://github.com/tvanasse/brainmap_structure_function_2020). Software packages including Nilearn (version 0.6.0b), Nibabel (version 2.5.1), and Nipype(version 1.4.2) were utilized.

For manuscripts utilizing custom algorithms or software that are central to the research but not yet described in published literature, software must be made available to editors and reviewers. We strongly encourage code deposition in a community repository (e.g. GitHub). See the Nature Research guidelines for submitting code & software for further information.

Data

Policy information about availability of data

All manuscripts must include a data availability statement. This statement should provide the following information, where applicable:

- Accession codes, unique identifiers, or web links for publicly available datasets
- A list of figures that have associated raw data
- A description of any restrictions on data availability

BrainMap data are publicly accessible at www.brainmap.org. ICA-computed component maps and average metadata component loading correlations can be downloaded at BrainMap's affiliated publication repository (http://brainmap.org/pubs/). Intermediate, per-experiment data formats used in ICA computation (modeled atrophy maps and modeled-activation maps) are available upon reasonable request via execution of a data-use agreement and with investigator support.

Field-specific reporting

Please select the one below that is the best fit for your research. If you are not sure, read the appropriate sections before making your selection.

☒ Life sciences ☐ Behavioural & social sciences ☐ Ecological, evolutionary & environmental sciences

For a reference copy of the document with all sections, see nature.com/documents/nr-reporting-summary-flat.pdf

Life sciences study design

All studies must disclose on these points even when the disclosure is negative.

Sample size	Two coordinate-based meta-analyses were performed using independent components analysis (ICA): 1) a functional ICA was performed on task-activation data; 2) a structural ICA was performed on voxel-based morphometry data. All data were drawn from the BrainMap database (www.brainmap.org) of published coordinate-reporting studies, using all available data with limited exclusions (see below). The functional ICA included N=7,865 experiments; the structural ICA included N=2,002 experiments.
Data exclusions	Data exclusions were made at the per-experiment level for redundancy (duplicate entries). Data exclusions were made at the per-category level for meta-data categories (either behavioral or by disease) containing < 10 experiments. Functional data utilized were limited to studies reporting on healthy control subjects, excluding task-activation studies in patient populations.
Replication	Independent components analysis was performed on two entirely distinct data sets (functional and structural) which were then compared for similarity. That is, the experimental design was a replication, testing whether structural pathology network architecture (a newer construct) recapitulated physiological network architecture (an established construct).
Randomization	Not applicable.
Blinding	Not applicable.

Reporting for specific materials, systems and methods

We require information from authors about some types of materials, experimental systems and methods used in many studies. Here, indicate whether each material, system or method listed is relevant to your study. If you are not sure if a list item applies to your research, read the appropriate section before selecting a response.

Materials & experimental systems

n/a	Involved in the study	
☒	☐	Antibodies
☒	☐	Eukaryotic cell lines
☒	☐	Palaeontology and archaeology
☒	☐	Animals and other organisms
☒	☐	Human research participants
☒	☐	Clinical data
☒	☐	Dual use research of concern
☒	☐	

Methods

n/a	Involved in the study	
☒	☐	ChIP-seq
☒	☐	Flow cytometry
☒	☐	MRI-based neuroimaging

his checklist template is licensed under a Creative Commons Attribution 4.0 International License, which permits use, sharing, adaptation, distribution and reproduction in any medium or format, as long as you ve appropriate credit to the original author(s) and the source, provide a link to the Creative Commons license, and indicate if changes were made. The images or other third party material in this article are cluded in the article's Creative Commons license, unless indicated otherwise in a credit line to the material. If material is not included in the article's Creative Commons license and your intended use is not ermitted by statutory regulation or exceeds the permitted use, you will need to obtain permission directly from the copyright holder. To view a copy of this license, visit tp://creativecommons.org/licenses/by/4.0/
